# Correction: Impact of bictegravir/emtricitabine/tenofovir alafenamide on health-related quality of life and economic outcomes in HIV care: Substudy of the BIC-NOW clinical trial

**DOI:** 10.1371/journal.pone.0341486

**Published:** 2026-01-20

**Authors:** Sergio Sequera-Arquelladas, María J. Vivancos, David Vinuesa, Antonio Collado, Ignacio De Los Santos, Patricia Sorni, Noemi Cabello-Clotet, Marta Montero, Carlos Ramos Font, Alberto Terron, Maria José Galindo, Onofre Martinez, Pablo Ryan, Mohamed Omar-Mohamed, Helena Albendín-Iglesias, Rosario Javier, Alberto Romero, Coral García Vallecillos, Miguel Ángel Calleja, Carmen Hidalgo-Tenorio

## Notice of Republication

This article was republished on on January 8, 2026 to remove confidential information. Please download this article again to view the correct version.
