# Supplementary material for: Impact of bictegravir/emtricitabine/tenofovir alafenamide on health-related quality of life and economic outcomes in HIV care: Substudy of the BIC-NOW clinical trial
Source: PLoS One. 2025 Sep 22;20(9):e0323167. doi: 10.1371/journal.pone.0323167 (PMC12453196; doi:10.1371/journal.pone.0323167)
Supplement: S1 File — (ZIP) [file pone.0323167.s001.zip › HIV_SI_Questionnaire_Translated (2).docx]

# HIV-SI Questionnaire

Please respond to the following questions considering the time period from when you began treatment until today:

| ITEM | YES | NO |
| --- | --- | --- |
| Have you noticed an increase in fatigue or loss of energy? |  |  |
| Have you had fever, chills, or sweats? |  |  |
| Have you felt dizzy or lightheaded? |  |  |
| Pain, numbness, or tingling in your hands or feet? |  |  |
| Memory problems or losses? |  |  |
| Have you experienced nausea or vomiting? |  |  |
| Diarrhea or loose or watery stools? |  |  |
| Do you feel more sad, down, or depressed than usual? |  |  |
| Do you feel nervous or anxious? |  |  |
| Do you have trouble falling asleep, or staying awake? |  |  |
| Skin problems? (e.g., dryness, itching...) |  |  |
| Cough or trouble catching your breath? |  |  |
| Have you had headaches? |  |  |
| Have you experienced loss of appetite or changes in taste? |  |  |
| Swelling, pain, or gas in your stomach? |  |  |
| Muscle or joint pain? |  |  |
| Issues with sexual activity? (e.g., loss of interest or lack of satisfaction) |  |  |
| Have you noticed body changes? (e.g., fat deposits or weight gain) |  |  |
| Weight loss issues? |  |  |
| Hair loss or changes in hair? |  |  |

Date of completion:
