# Supplementary material for: Impact of bictegravir/emtricitabine/tenofovir alafenamide on health-related quality of life and economic outcomes in HIV care: Substudy of the BIC-NOW clinical trial
Source: PLoS One. 2025 Sep 22;20(9):e0323167. doi: 10.1371/journal.pone.0323167 (PMC12453196; doi:10.1371/journal.pone.0323167)

CUESTIONARIO HIV-SI

Responde a las siguientes preguntas teniendo en cuenta el periodo de tiempo desde que iniciaste el tratamiento hasta hoy:

**ITEM**		**SI**		**NO**
¿Ha notado un incremento de fatiga o pérdida de energía?				
¿Ha tenido fiebre, escalofríos o sudores?				
¿Se ha notado mareado o aturdido?				
¿Dolor, adormecimiento u hormigueo en manos o pies?				
¿Problemas o pérdidas de memoria?				
¿Ha tenido nauseas o vómitos?				
¿Diarrea o deposiciones sueltas o líquidas?				
¿Se siente más triste, alicaído o deprimido de lo normal?				
¿Se siente nervioso o ansioso?				
¿Tiene dificultades para conciliar el sueño, o mantenerte despierto?				
¿Problemas de piel? (Ej. sequedad, picores...)				
¿Tos, o problemas para recuperar el aliento?				
¿Ha tenido dolores de cabeza?				
¿Ha experimentado pérdidas de apetito o alteraciones en el gusto?				
¿Hinchazón, dolor o gases de estómago?				
¿Dolor muscular o en las articulaciones?				
¿Problemas para mantener relaciones sexuales? (Ej. Pérdida de interés o falta de satisfacción)				
¿Ha notado cambios corporales? (Ej. Depósitos de grasa o ganancia de peso)				
¿Problemas de pérdida de peso?				
¿Pérdida de pelo o cambios en el pelo?				

Fecha de realización:

EQ - 5D

Cuestionario de Salud

Versión en español

(Spanish version)

Marque con una cruz como esta ☒ la afirmación en cada sección que describa mejor su estado de salud en el día de hoy.

Movilidad

No tengo problemas para caminar ☐

Tengo algunos problemas para caminar ☐

Tengo que estar en la cama ☐

Cuidado-Personal

No tengo problemas con el cuidado personal ☐

Tengo algunos problemas para lavarme o vestirme solo ☐

Soy incapaz de lavarme o vestirme solo ☐

Actividades de Todos los Días *(ej, trabajar, estudiar, hacer tareas domésticas, actividades familiares o realizadas durante el tiempo libre)*

No tengo problemas para realizar mis actividades de todos los días ☐

Tengo algunos problemas para realizar mis actividades de todos los días ☐

Soy incapaz de realizar mis actividades de todos los días ☐

Dolor/Malestar

No tengo dolor ni malestar ☐

Tengo moderado dolor o malestar ☐

Tengo mucho dolor o malestar ☐

Ansiedad/Depresión

No estoy ansioso/a ni deprimido/a ☐

Estoy moderadamente ansioso/a y/o deprimido/a ☐

Estoy extremadamente ansioso/a y/o deprimido/a ☐

Para ayudar a la gente a describir lo bueno o malo que es su estado de salud, hemos dibujado una escala parecida a un termómetro en el cual se marca con un 100 el mejor estado de salud que pueda imaginarse, y con un 0 el peor estado de salud que pueda imaginarse.

Por favor, dibuje una línea desde el cuadro que dice “su estado de salud hoy,” hasta el punto en la escala que, en su opinión, indique lo bueno o malo que es su estado de salud en el día de hoy.

SU ESTADO DE SALUD HOY

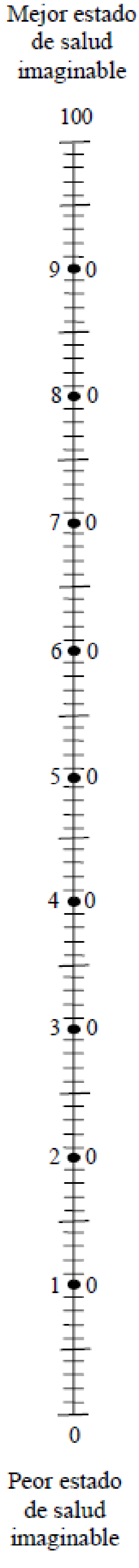

Supplement: S1 File — (ZIP) [file pone.0323167.s001.zip › Questionnaires (2).pdf]
