# Supplementary material for: Impact of bictegravir/emtricitabine/tenofovir alafenamide on health-related quality of life and economic outcomes in HIV care: Substudy of the BIC-NOW clinical trial
Source: PLoS One. 2025 Sep 22;20(9):e0323167. doi: 10.1371/journal.pone.0323167 (PMC12453196; doi:10.1371/journal.pone.0323167)
Supplement: S1 File — (ZIP) [file pone.0323167.s001.zip › S1 File. Supplementary appendixes.docx]

**Supplementary information: appendixes**

**Figure 1s.Comparative EQ-5D data**

**Table S1. Mean differences and 95% CI for EQ-5D variables**

| **Paired value** | **Mean diff** | **95% Confidence Interval of the Difference** | | **p-value** |
| --- | --- | --- | --- | --- |
|  |  | Lower | Upper |  |
| EQ5D-VAS BASELINE vs 24W | -0.573 | -2.610 | 1.464 | 0.58 |
| EQ5D-VAS BASELINE vs 48W | -1.557 | -3.671 | 0.557 | 0.15 |
| EQ5D-VAS BASELINE vs 48W - non AIDS | -1.670 | -2.970 | 0.030 | **.046*** |
| EQ5D-VAS under80 BASELINE vs 24W | 0.081 | 0.014 | 0.149 | **0.02** |
| EQ5D-VAS 80-89 BASELINE vs 24W | -0.054 | -0.142 | 0.034 | 0.23 |
| EQ5D-VA >=S BASELINE vs 24W | -0.027 | -0.110 | 0.056 | 0.52 |
| EQ5D-VAS under80 BASELINE vs 48W | 0.040 | -0.035 | 0.115 | 0.30 |
| EQ5D-VAS 80-89 BASELINE vs 48W | 0.011 | -0.074 | 0.097 | 0.79 |
| EQ5D-VAS >= BASELINE vs 48W | -0.051 | -0.140 | 0.038 | 0.26 |
| EQ5D-VAS 24W vs 48W | 0.388 | -1.190 | 1.967 | 0.63 |
| EQ5D-VAS under80 24W vs 48W | -0.065 | -0.120 | -0.010 | 0.02 |
| EQ5D-VAS 80-89 24W vs 48W | 0.065 | -0.023 | 0.152 | 0.15 |
| EQ5D-VAS >= 24W vs 48W | 0.000 | -0.084 | 0.084 | 1.00 |
| EQ-5D MOBILITY DIMENSION Baseline vs 48W | 0.026 | -0.018 | 0.070 | 0.25 |
| EQ-5D SELF-CARE DIMENSION Baseline vs 48W | 0.019 | -0.023 | 0.062 | 0.33 |
| EQ-5D USUAL ACTIVITIES DIMENSION Baseline vs 48W | 0.071 | 0.006 | 0.137 | **0.04** |
| EQ-5D PAIN OR DISCOMFORT DIMENSION Baseline vs 48W | 0.045 | -0.037 | 0.128 | 0.27 |
| EQ-5D ANXIETY OR DEPRESSION DIMENSION Baseline vs 48W | 0.065 | -0.016 | 0.146 | 0.11 |
| EQ5D UTILITYSCORE Baseline vs 48W | -0.023 | -0.045 | 0.000 | **0.01** |
| Profile (11111) Baseline vs W48 | -0.065 | -0.153 | 0.024 | 0.19 |
| Profile (11111) Baseline vs W48 - Non AIDS | -0.084 | -0.185 | 0.017 | **.031*** |

***: Exact Sig. (1-tailed);**

**Table S2. Mean differences and 95% CI for HIV-SI variables**

| **Paired value** | **Mean diff** | **95% Confidence Interval of the Difference** | | **p-value** |
| --- | --- | --- | --- | --- |
|  |  | Lower | Upper |  |
| HIV-SI: OVERALL NUMBER OF BOTHERSOME BASELINE vs W48 | 0.503 | -0.060 | 1.066 | **0.039*** |
| HIV-SI: OVERALL NUMBER OF BOTHERSOME BASELINE vs W24 | 0.308 | -0.132 | 0.748 | 0.169 |
| HIV-SI: OVERALL NUMBER OF BOTHERSOME W24 vs W48 | 0.102 | -0.334 | 0.538 | 0.645 |
| HIV-SI: Any BOTHERSOME symptom on BASELINE vs W48 | 0.132 | 0.052 | 0.211 | **0.001** |
| HIV-SI: Any BOTHERSOME symptom on BASELINE vs W24 | 0.049 | -0.026 | 0.125 | 0.199 |
| HIV-SI: Any BOTHERSOME symptom on W24 vs W48 | 0.084 | 0.008 | 0.160 | **0.030** |
| Fatigue/loss of energy, BASELINE vs 48W | 0.054 | -0.022 | 0.129 | 0.160 |
| Fevers/chills/sweats, BASELINE vs 48W | 0.024 | -0.032 | 0.079 | 0.395 |
| Dizzy/light headedness, BASELINE vs 48W | 0.018 | -0.044 | 0.080 | 0.565 |
| Pain/numbness/tingling in hands/feet, BASELINE vs 48W | -0.012 | -0.077 | 0.053 | 0.716 |
| Difficulty remembering, BASELINE vs 48W | -0.024 | -0.077 | 0.029 | 0.373 |
| Nausea/vomiting, BASELINE vs 48W | 0.030 | -0.019 | 0.079 | 0.226 |
| Diarrhea/loose bowels, BASELINE vs 48W | 0.072 | 0.008 | 0.136 | **0.043** |
| Sad/feeling down/depressed, BASELINE vs 48W | 0.048 | -0.034 | 0.130 | 0.249 |
| Nervous/anxious, BASELINE vs 48W | 0.072 | -0.004 | 0.148 | **0.044*** |
| Difficulty sleeping, BASELINE vs 48W | -0.012 | -0.091 | 0.067 | 0.764 |
| Skin problems/rash/itching, BASELINE vs 48W | 0.048 | -0.019 | 0.115 | 0.158 |
| Coughing/trouble breathing, BASELINE vs 48W | 0.024 | -0.023 | 0.071 | 0.319 |
| Headaches, BASELINE vs 48W | 0.030 | -0.042 | 0.102 | 0.413 |
| Loss of appetite, BASELINE vs 48W | 0.066 | 0.012 | 0.119 | **0.027** |
| Bloating/pain/gas in stomach, BASELINE vs 48W | 0.018 | -0.054 | 0.090 | 0.623 |
| Muscle aches/joint pain, BASELINE vs 48W | 0.018 | -0.052 | 0.088 | 0.614 |
| Problems with sex, BASELINE vs 48W | 0.048 | -0.017 | 0.112 | 0.145 |
| Changes in body composition, BASELINE vs 48W | 0.000 | -0.073 | 0.073 | 1.000 |
| Weight loss/wasting, BASELINE vs 48W | 0.012 | -0.041 | 0.065 | 0.656 |
| Hair loss/changes, BASELINE vs 48W | -0.030 | -0.094 | 0.034 | 0.355 |

***: Exact Sig. (1-tailed); HIV-SI: Human immunodeficiency virus symptom index**

**Details of sample size calculation:**

The sample size was calculated to obtain 90% power in a repeated-measures study (at baseline and given time point) using the following specific formula for this type of study design:

$$n=\left( \frac{Z_{\alpha/2}+Z_{\beta}}{d/{\sigma_{d}}} \right)^{2}$$

where the level of confidence (α) is0·05, corresponding to a critical Z_α_/2​ value of1·96; the statistical power (1−β) is90%, corresponding to a criticalZ_β_ value​of1·28.It was estimated that a single-group study designed to estimate HRQOL required around 193 patients to detec*t a minimal clinically important difference (MCID)* with 95% confidence level and 90% power, considering an MCID of 0·074,standard deviation of 0·3, and losses to the follow-up of 10%. This sample size allows significant differences in meanHRQOLscores to be estimated with the desired precision.In the substudy of participants in AIDS stage, it was estimated that a sample size of 40 patients would be sufficient to detect differences with 95% confidence interval and 85% power.

**Results of the Moses extreme reaction test (group category: AIDS stage yes/no)**

**General:**

**
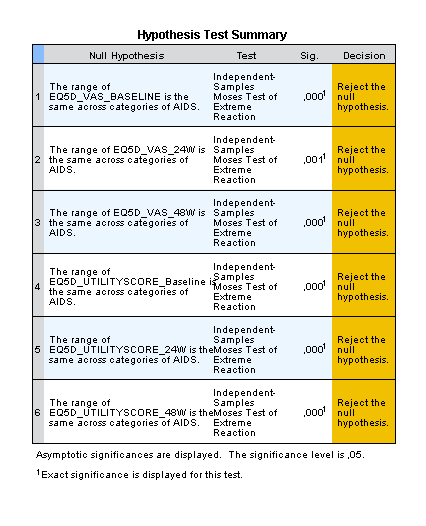
**

**EQ5D-VAS BASELINE:**

**Outliers trimmed from each end = 7**

**
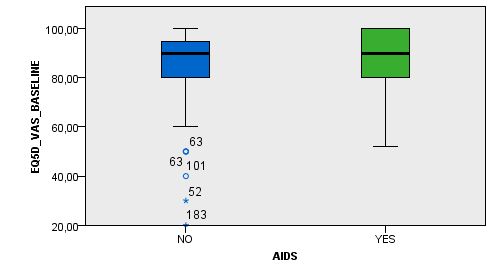
**

**EQ5D-VAS week 24:**

**Outliers trimmed from each end = 7**

**
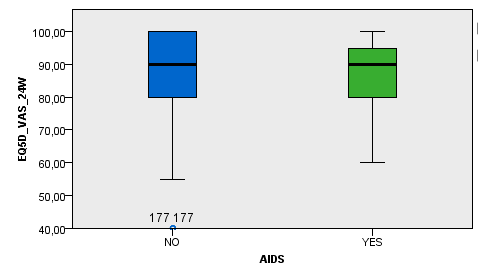
**

**EQ5D-VAS week 48:**

**Outliers trimmed from each end = 6**

**
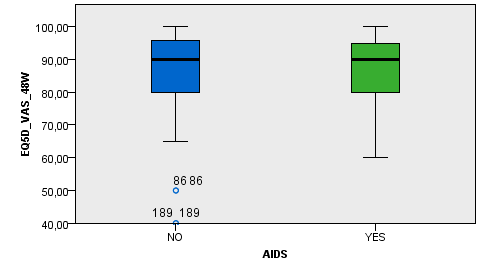
**

**EQ5D-Utility score baseline:**

**Outliers trimmed from each end = 7**

**
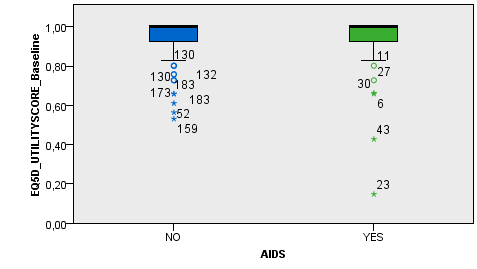
**

**EQ5D-Utility score week 24:**

**Outliers trimmed from each end = 6**

**
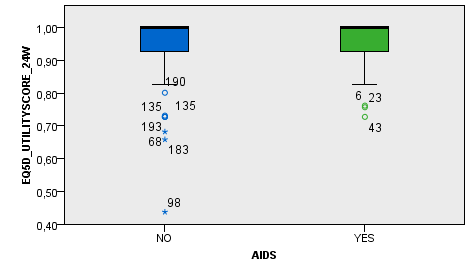
**

**EQ5D-Utility score week 48:**

**Outliers trimmed from each end = 6**

**
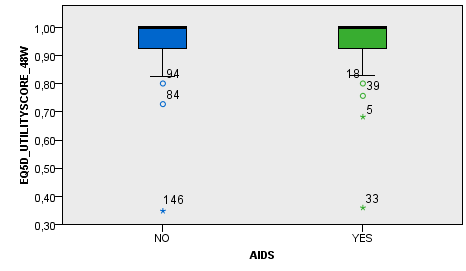
**

**REFERENCES**

1. Butler K, Anderson SJ, Hayward O, Jacob I, Punekar YS, Evitt LA, et al. Cost-effectiveness and budget impact of dolutegravir/lamivudine for treatment of human immunodeficiency virus (HIV-1) infection in the United States. J Manag Care Spec Pharm. 2021;27(7):891–903.

2. Rolle CP, Berhe M, Singh T, Ortiz R, Wurapa A, Ramgopal M, et al. Dolutegravir/lamivudine as a first-line regimen in a test-and-treat setting for newly diagnosed people living with HIV. AIDS. 2021;35(12):1957.

3. Hidalgo-Tenorio C, Sequera S, Vivancos MJ, Vinuesa D, Collado A, Santos IDL, et al. Bictegravir/emtricitabine/tenofovir alafenamide as first-line treatment in naïve HIV patients in a rapid-initiation model of care: BIC-NOW clinical trial. Int J Antimicrob Agents. 2024;107164.

4. Huhn GD, Crofoot G, Ramgopal M, Gathe J Jr, Bolan R, Luo D, et al. Darunavir/Cobicistat/Emtricitabine/Tenofovir Alafenamide in a Rapid-Initiation Model of Care for Human Immunodeficiency Virus Type 1 Infection: Primary Analysis of the DIAMOND Study. Clin Infect Dis. 2020;71(12):3110–7.
