# Supplementary material for: Impact of bictegravir/emtricitabine/tenofovir alafenamide on health-related quality of life and economic outcomes in HIV care: Substudy of the BIC-NOW clinical trial
Source: PLoS One. 2025 Sep 22;20(9):e0323167. doi: 10.1371/journal.pone.0323167 (PMC12453196; doi:10.1371/journal.pone.0323167)
Supplement: S2 Data — (PDF) [file pone.0323167.s003.pdf]

Real life study of Bictegravir, emtricitabine, and tenofovir alafenamide in HIV-1 infected treatment-naive patients using test and treat strategy. (BIC-NOW)

SPONSOR:
Fundación para la Investigación Biosanitaria de Andalucía Oriental - Alejandro Otero (FIBAO)

Principal Investigators:
Dra Carmen Hidalgo Tenorio (Universitary Hospital Virgen de las Nieves, Granada)

Approved by the Ethics Committee of Granada (CEImGranada) on 27 October 2020 (Act 13/20).

NCT number: NCT06177574

CONFIDENTIAL
This protocol contains information which is confidential and may also be legally privileged. It is for the exclusive use of the intended recipient(s). If you are not the intended recipient(s) please note that any form of disclosure, distribution, copying or use of this communication or the information in it or in any attachments is strictly prohibited and may be unlawful.

TABLE OF CONTENTS

TABLE OF CONTENTS..2
1. RESUMEN DEL PROTOCOLO EN CASTELLANO..3
2. BACKGROUND...6
3. SUMMARY..7
3.1 Title...7
3.2 Acronim...7
4. HYPOTHESYS...8
5. STUDY DESIGN...8
5.1 Overview of study design...8
5.2 Description of the treatment...8
5.3 Study interventions...8
5.4 Study population...8
5.5 Definitions...9
6. OBJECTIVES AND ENDPOINTS..9
7. SELECTION OF STUDY POPULATION..11
7.1 Inclusion criteria..11
7.2 Exclusion criteria..11
7.3 Subject procedence..11
8. SAMPLE SIZE CONSIDERATIONS:...11
9. STUDY DURATION..11
10. IMPLEMENTATION AND FEASIBILITY...12
11. PATIENT REPORTED OUTCOMES (PROS)..12
12. PHARMACOECONOMIC ANALYSIS...12
13. STATISTICAL ANALYSIS...13
14. SCHEDULE..13
15. PARTICIPATING CENTERS..14
16. BIBLIOGRAPHY...14

1. RESUMEN DEL PROTOCOLO EN CASTELLANO

La epidemia del VIH está lejos de ser erradicada. De acuerdo con los datos de la OMS y la UNAIDS de 2017, hay 36,9 millones de infectados en el mundo, 1,8 millones son nuevos diagnósticos, y el 10% de estos son niños menores de 15 años (1). En España hay mas de 10 diagnósticos al día y en la actualidad hay entre 140.000 y 170.000 personas infectadas por el VIH en España, desconociéndolo el 18%.(2)

La capacidad de transmisión del VIH está vinculada a la carga viral (CV) plasmática; y CV por debajo de 1.000 copias / mL de ARN-VIH tienen un riesgo significativamente menor de transmisión (3). En base a eso, hay un objetivo para el año 2020 llamado 90-90-90: un objetivo de tratamiento ambicioso para ayudar a poner fin a la epidemia de sida (para 2020, el 90% de todas las personas que viven con el VIH conocerán su estado de VIH; para 2020, 90 El% de todas las personas con infección por VIH diagnosticada recibirán terapia antirretroviral sostenida; para 2020, el 90% de todas las personas que reciban terapia antirretroviral tendrán supresión viral).

Con los antirretrovirales clásicos, la epidemia de VIH sigue creciendo y comenzaron nuevas terapias como la "profilaxis pre-exposición" (el uso de medicamentos antirretrovirales como una forma de prevenir nuevas infecciones entre las personas con mayor riesgo) con Tenofovir Disoproxil Fumarate (TDF), pero los ensayos clínicos han resultados mixtos dependiendo del nivel de adherencia (4). Otra estrategia fue el inicio rápido de TAR para aquellos que dieron positivo, llamado prueba y tratamiento (Test&treat); demostrando que una reducción en el tiempo desde el diagnóstico hasta el inicio del tratamiento resultó en tasas más altas de supresión viral (5) y una mejor adherencia al sistema (6).

Los inhibidores de la transferencia de la cadena de integrasa (INSTI) son un grupo de ARV, y los ensayos clínicos muestran que los regímenes basados en INSTI dan como resultado una supresión virológica más rápida en comparación con otros grupos (7) siendo una gran opción para la estrategia de tratamiento rápido.

Bictegravir (BIC) es un INSTI comercializado en España en un régimen de tableta única (STR) asociado con tenofovir alafenamida / emtricitabina (TAF / FTC); tiene una gran barrera genética (por lo que no necesita una prueba de resistencia genotípica previa para ser recetada) y no experimenta diferencias en la efectividad del género, raza, edad, recuento de CD4 o carga viral plasmática; no es necesario realizar un estudio de HLA y solo se necesita prescribir creatinina (no se recomienda su uso en pacientes con CrCl <30 ml / minuto) (8)

Hasta ahora, el único TAR capaz de eso era TAF / FTC / Darunavir / cobicistat, demostrado en el ensayo clínico DIAMOND (9)

Los informes elaborados por el paciente (PROs) se han considerado durante mucho tiempo como herramientas importantes en la evaluación y diferenciación de las estrategias de

tratamiento, cuando se utilizan para medir el efecto del TAR en uno o más conceptos (como la calidad de vida relacionada con la salud (CVRS) o los síntomas) (10) Además, para los sujetos sin tratamiento previo, el uso de PRO ofrece la oportunidad de evaluar el TAR desde el inicio del tratamiento, proporcionando información sobre cómo el TAR puede alterar los resultados de la calidad de vida y, por lo tanto, una mejor comprensión de esta relación.

Hay un interés creciente en la importancia de evaluar la implementación y la viabilidad de nuevas estrategias de tratamiento, en términos de aceptación por parte de los profesionales de la salud y el conocimiento médico necesario para la implementación (11). Implementación, conocida como la introducción de una innovación en las rutinas diarias; y la viabilidad, definida como la medida en que una nueva estrategia puede llevarse a cabo con éxito dentro de un entorno determinado, puede evaluarse considerando la aceptabilidad de la estrategia entre los profesionales de la salud y los costos de integrarla en entornos clínicos.

Por todas las razones anteriores, el objetivo principal de este estudio es analizar en pacientes con VIH sin tratamiento previo la actividad antiviral, utilizando una estrategia de prueba y tratamiento (Test&treat), en la vida real de BIC / FTC / TAF. En segundo lugar, este estudio tiene como objetivo evaluar los resultados para la implementación de la estrategia de prueba y tratamiento (Test&treat) basada en la evidencia evaluando: [1] la aceptabilidad (adherencia y retención de medicamentos en la atención) de la estrategia de prueba y tratamiento (Test&treat) para pacientes con VIH sin tratamiento previo, [2] el efecto de la estrategia de prueba y tratamiento (Test&treat) sobre la calidad de vida relacionada con la salud (EQ-5D genérico) y los síntomas relacionados con el VIH (VIH-SI dicotomizado) a pacientes con VIH sin tratamiento previo a través de Resultados informados por el paciente (PRO), [3] el impacto económico de esto ART mediante diversos análisis farmacoeconómicos, [4] la viabilidad de la estrategia de prueba y tratamiento (Test&treat) en entornos de salud pública españoles y [5] la viabilidad general de la implementación. Los objetivos clínicos secundarios son: Evaluar el efecto de la demografía del paciente y las características basales en la respuesta a BIC / FTC / TAF a lo largo del tiempo, evaluar las pruebas de resistencia viral en el sujeto que cumple con la falla virológica confirmada, evaluar la actividad antiviral, evaluar la seguridad y la tolerabilidad de BIC / FTC / TAF a lo largo del tiempo, para analizar la adherencia del sujeto al sistema de salud.

RESUMEN DE OBJETIVOS Y METODOLOGÍA DEL PROYECTO

Objetivo Principal: Analizar la actividad antiviral de BIC / FTC / TAF a las 24 y 48 semanas en sujetos con infección por VIH-1, sin ART previo, utilizando la estrategia de prueba y tratamiento (Test&treat).

Pacientes y métodos: Este es un estudio de fase IV, observacional, prospectivo, de un solo brazo, multicéntrico y abierto de 48 semanas. El estudio se llevará a cabo en

aproximadamente 139 sujetos sin tratamiento previo infectados con VIH-1 que comenzaron su TAR con una terapia de BIC / FTC / TAF en una estrategia de prueba y tratamiento (Test&treat). Los sujetos se inscribirán después de firmar el consentimiento informado.

Criterios de inclusión:

1. Adultos infectados por VIH-1 (> 17 años)
2. Sin TAR previo.
3. Ser capaz de cumplir con los requisitos e instrucciones del protocolo.
4. El sujeto, o el representante del sujeto, debe de dar su consentimiento informado firmado.

Criterios de exclusión:

1. Mujeres que planean quedar embarazadas durante el periodo de estudio.
2. Pacientes que, a juicio del investigador, presentan un riesgo de abandono significativo o una esperanza de vida inferior al final del estudio.
3. Los pacientes con necesidad anticipada de cambiar el TAR antes de finalizar el estudio.

Procedencia del sujeto:

Los sujetos serán reclutados en unidades de enfermedades infecciosas de varios centros de salud públicos españoles.

Procedimientos:

Las visitas de seguimiento se mantendrán en sus centros de salud habituales. En la visita basal (V0), el personal del sitio de estudio obtendrá el consentimiento informado por escrito de cada sujeto potencialmente elegible (o su representante legal) antes de iniciar cualquier procedimiento.

Después de firmar el consentimiento informado, los sujetos completarán los procedimientos basales.

En las visitas en la semana 4 (V1), 24 (V2) y 48 (V3) se recogerán las variables clínicas. También se solicitará por vía oral que se clasifiquen y registren los eventos adversos en cada visita.

En cada visita, los sujetos completarán dos módulos de PRO: EQ-5D y HIV-SI.

2. BACKGROUND

The HIV epidemic is not over, according to data of WHO and UNAIDS in 2017, a total of 36.9 million people infected with HIV in the world, 1,8 million people were newly infected with HIV, and 10% of those among children <15 years old (1). In Spain there are more than 10 new infections diagnosed every day, a 0,2% of world total; with 3381 new infections and a total of 140.000-170.000 are living in Spain with HIV at 2017, 18% of those do not knowing it. In the province of Andalusia, results are better but still high with 574 new infections in 2017 (6.85/100.000 pp) (2)

The capability of transmission of the HIV is linked to the plasmatic viral load; and numbers under 1.000 copies/mL of RNA-HIV have significantly less risk of transmission (3). Based on that, there is an objective to year 2020 called 90-90-90: an ambitious treatment target to help end the aids epidemic (By 2020, 90% of all people living with HIV will know their HIV status; By 2020, 90% of all people with diagnosed HIV infection will receive sustained antiretroviral therapy; By 2020, 90% of all people receiving antiretroviral therapy will have viral suppression).

With the classical ARTs the HIV epidemic keep growing and new therapies began such as "pre-exposure prophylaxis" (using antiretroviral medications as a way to prevent new infections among those at greatest risk) with Tenofovir Disoproxil Fumarate (TDF), but clinical trials have mixed results depending upon the level of adherence (4). Another strategy was the rapid ART initiation for those who test positive, called *test and treat*; demonstrating that a reduction in time from diagnosis to treatment initiation resulted in higher rates of viral suppression (5), and better adherence to the system (6).

Integrase strand transfer inhibitors (INSTIs) are a group of ARVs, and clinical trials show that INSTI based regimens result in more rapid virologic suppression compared to other groups (7) being a great choice for the rapid treatment strategy.

Bictegravir (BIC) is an INSTI commercialized is Spain in a single tablet regimen (STR) associated with tenofovir alafenamide/emtricitabine (TAF/FTC); have a great genetic barrier (so do not need a previous genotypic resistance test to be prescribed) and does not experience differences in effectivity from gender, race, age, CD4 count or plasmatic viral load; no need of HLA study and only creatinine is needed to be prescribed (use is not recommended in patients with CrCl <30 mL/minute) (8)

Until now, the only ART capable of that was TAF/FTC/Darunavir/cobicistat, demonstrated in the DIAMOND clinical trial (9)

Patient Reported Outcomes (PROs) have long been regarded as important tools in the evaluation and differentiation of treatment strategies, when used to measure the effect of the ART on one or more concepts (such as Health Related Quality of Life (HRQL) or

symptoms) (10). Additionally, for treatment-naïve subjects, the use of PROs bring up the opportunity to assess the ART since the initiation of the treatment, providing information of how the ART may alter quality of life outcomes and therefore a better understanding of this relationship .

There is a growing emphasis in the importance of evaluating implementation and feasibility of new treatment strategies, in terms of acceptance by healthcare professionals and physician knowledge needed for implementation (11). Implementation, referred to as the introduction of an innovation in daily routines; and feasibility, defined as the extent to which a new strategy can be successfully carried out within a given setting, can be assessed by considering the acceptability of the strategy among the healthcare professionals and the costs of integrating it into clinical settings.

For all the reasons above, the primary objective of this study is to analyze in treatment-naïve HIV patients the antiviral activity, using a test and treat strategy, in real life of BIC/FTC/TAF. Secondary, this study aims to evaluate outcomes for implementation of the evidence based test and treat strategy assessing: [1] the acceptability (Medication adherence and retention in care) of test and treat strategy to treatment-naïve HIV patients, [2] the effect of test and treat strategy on health related quality of life (Generic EQ-5D) and HIV-related symptoms (dichotomized HIV-SI) to treatment-naïve HIV patients through Patient Reported Outcomes (PROs), [3] the economic impact of this ART by various pharmaco-economic analyses, [4] the feasibility of test and treat strategy in Spanish public healthcare settings and [5] the overall feasibility of implementation. Secondary clinical objectives are: To evaluate the effect of patient demographics and baseline characteristics on response to BIC/FTC/TAF over time, to asses viral resistance tests in subject meeting confirmed virologic failure, to evaluate antiviral activity, to evaluate the safety and tolerability of BIC/FTC/TAF over time, to analyze subject adherence to the healthcare system.

3. SUMMARY

3.1 Title

Real life study of BIC/FTC/TAF in HIV-1 infected treatment-naive patients using test and treat strategy. (BIC-NOW)

3.2 Acronim

BIC-NOW

4. **HYPOTHESYS**

This study is designed to provide real world data about the effectiveness of BIC/FTC/TAF in a sample of treatment-naïve HIV patients using a test and treat strategy. With this data the study will show that this therapy is safe and effective compared with previous data from different treatments.

5. **STUDY DESIGN**

5.1 Overview of study design

This is a phase IV, observational, prospective, single-arm, multicentre, and open 48-week study. The study will be conducted in approximately 139 HIV-1 infected, treatment-naive subjects who began their ART with a therapy of BIC/FTC/TAF in a strategy of test and treat. The subjects will be enrolled after they sign the informed consent.
Week 24 interim analysis: Interim analyses will be performed once all patients continuing treatment with BIC/FTC/TAF reach week 24.

5.2 Description of the treatment

The election to receive the BIC/FTC/TAF regimen will be made according to standard clinical practice, before and independently of their inclusion in the study.
Drugs the subjects are using before their inclusion in the study will be maintained according to medical criteria in the clinical practice.

5.3 Study interventions

The follow-up visits will be maintained at their usual healthcare centers.
At the basal visit (V0) written informed consent will be obtained from each potentially elegible subject (or his/her legal representative) by study site personnel prior to the initiation of any procedure.
After signing an informed consent, subjects will complete the basal procedures, which are:
Anthropometric measures (high, weight, BMI and waist line) and a blood sample before the first dose of the therapy (to measure clinical chemistry, haematology, fasting lipids, quantitative plasma HIV-1-RNA, lymphocyte subset, HBsAg, anti-HBc, anti-HBs, HBV DNA, HIV genotyping for resistance).
In visits at week 4 (V1), 24 (V2) and 48(V3) anthropometric measures (high, weight, BMI and waist line) and a blood sample before the first dose of the ART (to measure clinical chemistry, haematology, fasting lipids, quantitative plasma HIV-1-RNA, lymphocyte subset, HBsAg, anti-HBc, anti-HBs, HBV DNA) will be recorded. Also adverse events will be asked orally to be classified and recorded at each visit.
In each visit, subjects will complete two modules of PROs: EQ-5D and HIV-SI.
In case of a confirmed virological failure, a genotypic resistance test will be done.
Patients who drops or lost to follow ups will be recorded during the study.

5.4 Study population

The study will be conducted in approximately 139 HIV-1 infected, ART-naive adults who began their ART with BIC/FTC/TAF in a test and treat strategy, without limits of viral load or CD4 lymphocytes recount at screening.

The following population will be assessed:

Intent-to-treat (ITT) Population: This population will consist of all randomized subjects who receive at least one dose of study medication.

Intent-to-treat modified (ITT-m) Population: this population will consist of subjects in the ITT Population with the exception of mild protocol violators, those who discontinue for reasons other than those related to treatment (such as adverse events, tolerability or lack of efficacy).

Per protocol (PP) Population: This population will consist of subjects in the ITT Population with exception of major protocol violators, such as violations which could affect the assessment of antiviral activity.

5.5 Definitions

- Effectivity: Plasma HIV-1 RNA <50 copies/mL at week 48 using the FDA Sanpshot algorithm for ITT, ITT-m and PP populations.
- Virological failure: Two consecutive viral loads >50 copies/mL, after achieving virological suppression. Failure to achieve viral suppression after 24 weeks will also be considered a virological failure.
- Deferred treatment: ART initiated two weeks after being attended at the hospital.
- "Test and treat" strategy, fast/immediate treatment: We will differentiate the immediate treatment, which will consist of treatments initiated the same day the subject came to baseline visit (first visit with the infectious diseases specialist) but without baseline laboratory information; from fast treatment, which will consist of treatments initiated within two weeks from their baseline visit but still without baseline laboratory information.
- Patient Reported Ourcomes (PROs): any report of the status of a patient's health condition that comes directly from the patient without interpretation of the patient's response by a clinician or anyone else.
- Drop out: subjects who prefer to discontinue their therapy for reasons other than those related to treatment.
- Adherence: will be analyzed as lost to follow up, percentage of patients who drop out at week 48.
- Adverse Events: Grade of AE will be evaluated using the "DAIDS AE Grading Table" (12) which is a descriptive terminology which can be utilized for Adverse Event (AE) reporting. A grading (severity) scale is provided for each AE term.
- **The investigator, or designee, has a legal responsibility to notify every severe adverse event to the country specific regulatory authority (AEMPS) via Eudravigilance_CTM as indicated at section "¿Deben notificarse al CEIm las sospechas de reacciones adversas graves e inesperada?" into the document "instrucciones de la Agencia Española del Medicamente y Producto Sanitario para la realización de ensayos clínicos en España.**

6. OBJECTIVES AND ENDPOINTS

5.1. Primary objective

1. To analyze antiviral activity of BIC/FTC/TAF at 24 and 48 weeks in HIV-1-infected, ART-naive subjects, using test and treat strategy.
   a. Endpoint: Proportion of subjects with plasma HIV-1 RNA <50 copies/mL at week 24 using FDA Snapshot algorithm.

    b. Endpoint: Proportion of subjects with plasma HIV-1 RNA <50 copies/mL at week 48 using FDA Snapshot algorithm.

5.2 Secondary implementation objectives

Implementation variables must provide data to answer the questions about acceptability, costs impact, on-patient effects and feasibility of the implemented strategy among others (13). Based on this background, we formulated the following implementation objectives:

1. To evaluate test and treat strategy acceptability among treatment-naïve HIV patients assessing medication adherence and retention in care:
    a. Medication adherence endpoint: Number and proportion of pills taken, self-reported at follow-up visits paired with pharmacy dispensing information.
    b. Retention in care endpoint: Proportion of subject completing all study visits.
2. To measure the effect of test and treat strategy on health related quality of life and HIV-related symptoms to treatment-naïve HIV patients through PROs EQ-5D and dichotomized HIV-SI.
3. To evaluate the costs of this strategy, analyzing the economic impact of this ART assessed by various pharmaco-economic analyses (see point 11 below for details).
4. To assess the feasibility of test and treat strategy among treatment-naïve HIV patients, analyzing if this strategy could be successfully carried out within our settings.
    a. Endpoint: Time to full enrolment of 139 eligible treatment-naïve HIV patients at 22 public healthcare centers in Spain and by physicians using screening data reports.
5. To evaluate the overall feasibility of the test and treat strategy, taking as factors the previous analysis of acceptability, effects of the strategy on health related quality of life and HIV-related symptoms, economic impact and the feasibility of the strategy among treatment-naïve HIV patients.

5.3 Secondary clinical objectives

1. To evaluate the effect of patient demographics and baseline characteristics on response to BIC/FTC/TAF over time.
    a. Endpoint: Proportion of subjects by patient subgroup (e.g. by gender, age…) with plasma HIV-1 RNA <50 copies/mL at week 24 and 48 using FDA Snapshot algorithm.
    b. Endpoint: Change from baseline lymphocyte count at week 24 and 48 by patient subgroup.
2. To asses viral resistance tests in subject meeting confirmed virologic failure.
    a. Endpoint: Incidence of treatment-emergent genotypic or/and phenotypic resistence to any ART.
3. To evaluate antiviral activity.
    a. Endpoint: Time to viral suppression and absolute changes from baseline in lymphocyte count at week 24 and 48.
4. To evaluate the safety and tolerability of BIC/FTC/TAF over time.

a. Endpoint: Incidence and severity of adverse events and laboratory abnormalities.

7. SELECTION OF STUDY POPULATION

Deviations from inclusion and exclusion criteria are not allowed to maintain the integrity of the study.

7.1 Inclusion criteria

1. HIV-1 infected adults (>17 y.o.)
2. Antiretroviral-naïve.
3. Be able to comply with protocol requirements and instructions.
4. Subject or the subject's representative capable of giving signed informed consent.

7.2 Exclusion criteria

1. Women who are breastfeeding or plan to become pregnant during the study.
2. Patients who in the investigator's judgment, poses a significant drop out risk or life expectancy inferior to study ending.
3. Patients with anticipated need to change the ART before study ending.

7.3 Subject procedence

Subjects will be recruited at infectious diseases units from various Spanish public healthcare centers.

8. SAMPLE SIZE CONSIDERATIONS:

Clinical trials shows efficacy for BIC/FTC/TAF combination of 92% in ITT population and FDA Snapshot algorithm. With a confidence interval of 95%, precision of 5% and assuming 10% of drop outs we estimate the number of subjects in 139.

9. STUDY DURATION

The study will include a follow-up of 48 weeks to each subject since their inclusion.

10. IMPLEMENTATION AND FEASIBILITY

To evaluate the different outcomes of implementation and feasibility, we are going to asses:

1. Test and treat strategy acceptability (medication adherence and retention in care) to treatment-naïve HIV patients.
    a. Medication Adherence will be assessed by the number and proportion of pills taken, self-reported at follow-up visits paired with pharmacy dispensing information.
    b. Retention in care will be assessed by proportion of subject completing all study visits.
2. The effect of test and treat strategy on health related quality of life and HIV-related symptoms to treatment-naïve HIV patients.
    a. This will be assessed by Patient Reported Outcomes (PROs), using a multidimensional generic Health Related Quality of Life instrument (EQ-5D) and a specific one to measure HIV-related symptoms (dichotomized HIV-SI).
3. The economic impact of this ART.
    a. The economic impact will be assessed by various pharmaco-economic analyses (minimization of costs study, a cost-effectivity ratio analysis and an incremental cost-effectivity ratio will be realized) evaluating our results with those from previous literature of other popular ART choices.
4. Feasibility of test and treat strategy among treatment-naïve HIV patients at 22 health centers in Spain.
    a. Feasibility will be assessed by time to full enrolment of 139 eligible treatment-naïve HIV patients and by physicians using screening data reports.
5. Overall feasibility of test and treat implementation.
    a. Overall feasibility will be assessed after evaluating the previous implementation outcomes.

11. PATIENT REPORTED OUTCOMES (PROS)

A planned implementation analysis of the effect of test and treat strategy with BIC/FTC/TAF on health related quality of life and HIV- related symptoms to treatment-naïve patients through patient-reported outcomes was conducted for the study. In the study, the HIV-related symptoms module (HIV-SI) and a multidimensional generic Health Related Quality of Life module (EQ-5D) are going to be administrated at each visit. The HIV-SI responses to each of the 20 items were dichotomized as bothersome or not bothersome.

12. PHARMACOECONOMIC ANALYSIS

In the pharmacoeconomic analysis, the BIC/FTC/TAF regimen will be compared with other test and treat treatments such as TAF/FTC/Darunavir/cobicistat as reference treatment.

Effectiveness data based on an intention-to-treat (ITT) analysis will be obtained for BIC/FTC/TAF from the present study, for TAF/FTC/Darunavir/cobicistat from the DIAMOND trial, (9) (or a most recent trial) which is a clinical trial in patients with a test and minimization analysis to estimate the saving that would result from the adoption of BIC/FTC/TAF instead of the other therapies. We will also carry out a cost- effectiveness

ratio analysis, dividing the cost of each treatment by its effectiveness for virological suppression. The incremental cost- effectiveness ratio will be determined by dividing the cost differences by the effectiveness increase.

13. STATISTICAL ANALYSIS

Descriptive analyses:
Descriptions of the principal variables collected in the study were, for the quantitative variables, measures of central tendencies and dispersion: mean, standard deviation, median, percentiles, and for the qualitative variables, absolute and relative frequencies.

To assess the effectivity, subjects response with VL<50copies/mL will be analyzed.

Effectivity results will be compared among patients classified as immediate treatment and those classified as fast treatment.

At least three analyses will be performed to assess endpoints when every subject completed the week 4, 24 and 48, being the primary analysis at week 48.

An interim analysis will be performed once all patients continuing treatment with BIC/FTC/TAF reach week 24.

Data will be recorded from their clinical history to a standard database, using IBM-SPSS:

- Demographics: Age, gender, height, weight, BMI and waist.
- Full blood haemogram and chemistry: Creatinin, urea, CKD-EPI, GOT, GPT, GGT, FA, Calcium, Phosphorus, total cholesterol, HDL, LDL, TC/HDL, lymphocytes nadir and counts, ratio CD4/CD8 and viral load. At v0, v1, v2 and v3.
- Serology: VHB, VHA, IgG, VHC, CMV IgG, TXP IgG. At V0, V1, V2 and V3.
- Adverse events record: at V1, V2 and V3.

This data will be monitorized remotely from the coordinator center.

14. SCHEDULE

Estimated schedule:
- Month 0: open recruitment.
- Month 12: closed recruitment.
- Month 18: Communication with preliminary week 24 data submitted to national and international HIV congress.
- Month 24: Last visit, last patient. Participating center may send their data to coordinator center. (Hospital Universitario Virgen de las Nieves, Granada).
- Month 25: Data analysis, article elaboration and submission to journals with IF Q1-Q2.

15. PARTICIPATING CENTERS

Marta Montero	HU la Fé, Valencia
Mª Jesus Vivancos	H Ramón y Cajal, Madrid
Onofre Martínez	H Santa Lucía, Cartagena
María Arrizabalaga	HU Son Llatzer, Baleares
Pablo Ryan	HU Infanta Leonor, Madrid
Ignacio de los Santos	HU de la Princesa, Madrid
Antonio Collado	HU torrecárdenas, Almería
Carlos Galera	HU Virgen de la Arrixaca, Murcia
David Vinuesa	HU San Cecilio, Granada
Omar Mohamed	H Jaén, Jaén
Ana López	HU Canarias, Canarias
Vicente estrada	Hospital Clínico San Carlos, MADRID
Alberto de santiago	Hospital Universitario Puerta De Hierro, MADRID
Aterron	Hospital de Jerez
Carmen Hidalgo	Hospital Universitario Virgen de las Nieves

16. BIBLIOGRAPHY

1. Global HIV & AIDS statistics — 2018 fact sheet 2019 [Available from: https://www.unaids.org/en/resources/fact-sheet].
2. Ministerio de Sanidad Servicios Sociales e Igualdad. VIGILANCIA EPIDEMIOLÓGICA DEL VIH y SIDA EN ESPAÑA 2017. ACTUALIZACIÓN 30 de junio de 2018. [Available from: http://www.isciii.es/ISCIII/es/contenidos/fd-servicios-cientifico-tecnicos/fd-vigilancias-alertas/fd-enfermedades/fd-sida/pdf_sida/Informe_VIH_SIDA_2018_21112018.pdf]
3. Hughes JP et al. Determinants of Per-Coital-Act HIV-1 Infectivity Among African HIV-1-Serodiscordant Couples. J Infect Dis. 205 (3): 358-365, 2012
4. Grant RM, Anderson PL, McMahan V, Liu A, Amico KR, Mehrotra M, et al. Uptake of pre-exposure prophylaxis, sexual practices, and HIV incidence in men and transgender women who have sex with men: a cohort study. Lancet Infect Dis. 2014;14(9):820-9.
5. Havlir D, Charlebois E, Balzer L, et al. SEARCH community cluster randomized study of HIV "test and treat" using multi- disease approach and streamlined care in rural Uganda and Kenya. Presented at the 22nd International AIDS Conference, Amsterdam, the Netherlands. July 2018. http://programme.aids2018.org/Abstract/Abstract/13469 (Accessed on August 03, 2018).

6. Das M, Christopoulos KA, Geckeler D, Huriaux E, Cohen SE, Philip S, et al. Linkage to HIV Care in San Francisco: Implications of Measure Selection. J Acquir Immune Defic Syndr. 2013;64(0 1):S27-32.
7. Jacobson K, Ogbuagu O. Integrase inhibitor-based regimens result in more rapid virologic suppression rates among treatment-naïve human immunodeficiency virus–infected patients compared to non-nucleoside and protease inhibitor–based regimens in a real-world clinical setting: A retrospective cohort study. Medicine (Baltimore). 972018.
8. Gallant J, Lazzarin A, Mills A, Orkin C, Podzamczer D, Tebas P, et al. Bictegravir, emtricitabine, and tenofovir alafenamide versus dolutegravir, abacavir, and lamivudine for initial treatment of HIV-1 infection (GS-US-380-1489): a double-blind, multicentre, phase 3, randomised controlled non-inferiority trial. Lancet 2017;390:2063-72. 10.1016/S0140-6736(17)32299-7
9. Huhn G, Crofoot G, Ramgopal M, et al. Darunavir/cobicistat/emtricitabine/tenofovir alafenamide (D/C/F/TAF) rapid initiation for HIV-1 infection: primary analysis of the DIAMOND study. Poster presented at: 13th Annual American Conference for the Treatment of HIV (ACTHIV); April 11-13, 2019; Miami, Florida.
10. Simpson KN, Hanson KA, Harding G, Haider S, Tawadrous M, Khachatryan A, et al. Patient reported outcome instruments used in clinical trials of HIV-infected adults on NNRTI-based therapy: a 10-year review. Health Qual Life Outcomes. 2013;11:164.
11. Richards D, Hallberg I, editors. Complex interventions in health: an overview of research methods. Oxford and New York: Routledge; 2015.
12. U.S. Department of Health and Human Services, National Institutes of Health, National Institute of Allergy and Infectious Diseases, Division of AIDS. Division of AIDS (DAIDS) Table for Grading the Severity of Adult and Pediatric Adverse Events, Corrected Version 2.1. [July 2017]. Available from: https://rsc.niaid.nih.gov/sites/default/files/daidsgradingcorrectedv21.pdf
13. Proctor E, Silmere H, Raghavan R, Hovmand P, Aarons G, Bunger A, Griffey R, Hensley M, 2011. Outcomes for implementation research: conceptual distinctions, measurement challenges, and research agenda. Adm. Policy Ment. Health 38, 65–76. 10.1007/s10488-010-0319-7.
