# Supplementary material for: Impact of bictegravir/emtricitabine/tenofovir alafenamide on health-related quality of life and economic outcomes in HIV care: Substudy of the BIC-NOW clinical trial
Source: PLoS One. 2025 Sep 22;20(9):e0323167. doi: 10.1371/journal.pone.0323167 (PMC12453196; doi:10.1371/journal.pone.0323167)
Supplement: Attachment — Submitted filename: Response_to_Reviewers_auresp_2.docx [file pone.0323167.s005.docx]

Reviewer #1: Thank you for your careful consideration of my comments. There are still a couple comments where I continue to disagree.

First to this response:

"As stated above, we always used the same patient database (all participants in the BICNOW clinical trial). However, as expressly documented, not all participants remained throughout the study, and some questionnaires were invalid. We analyzed all valid questionnaires available at each time point, and their number is reported in the tables. In this way, we believe that our study offers a robust and transparent analysis of real-world changes in the quality of life of these patients."

In table 2, I presume your p-value is for the 169 participants who have week 48 observations. If so, what are the numbers that are going into that test? The week 48 value is, but the baseline is not because you have presented the value with n=186. That thinking suggests that the way you have presented table 2 is not transparent. Regarding your comment about robustness, I'm not sure I follow in a statistical sense since, presumably the 18 participants (186-169) don't contribute to the statistical analyses.

We thank the reviewer for this comment. We have addressed this concern by incorporating data in revised Tables 2 and 3 on the number of observations considered at each time point for the p-value calculation.

**Table 2. EQ-5D-3L – results per evaluated dimension**

|  | **BASELINE**  **N =186** | **24W**  **N=178** | **48W**  **N=169** | **p-value**  **(BASELINE vs 48W)**  **N=169** |
| --- | --- | --- | --- | --- |

**Table 3. Results of dichotomized HIV-SI questionnaire (bothersome/non-bothersome)**

|  | **BASELINE**  **N = 199** | **W24**  **N= 187** | **W48**  **N=172** | **p-value**  **(BASELINE vs 48W)**  **N=172** |
| --- | --- | --- | --- | --- |

Second, regarding this response to my comment about Kolmogorv-Smirnov:

"We are grateful for this comment, which will be considered in future studies. On the other hand, this is the most frequently applied test in clinical research and is maintained in the present investigation."

If we as scientists followed this logic, we would never change our analytic methods. This doesn't appear to be a valid scientific reason, so I still encourage you to make this change.

As a result of this encouragement, we applied both tests proposed by the reviewer using version 4.4.3 of the R statistical package. There were no differences in normality results among the three tests (Kolmogorov-Smirnov,Cramer-von Mises,and Anderson-Darling tests). Following the reviewer’s suggestion, we selected the Cramer-von Mises test for the revised manuscript.
